# Valproate Sodium Protects Blood Brain Barrier Integrity in Intracerebral Hemorrhage Mice

**DOI:** 10.1155/2020/8884320

**Published:** 2020-11-10

**Authors:** Wei Zhao, Lianhua Zhao, Zaiyu Guo, Yanwei Hou, Jiafeng Jiang, Yijun Song

**Affiliations:** ^1^General Medicine Department, Tianjin Medical University General Hospital, Tianjin, China; ^2^Department of Neurology, Tianjin TEDA Hospital, Tianjin, China; ^3^Tianjin Neurological Institute, Tianjin, China

## Abstract

Valproate sodium (VPA) is a traditional antiepileptic drug with a neuroprotective role in cerebrovascular disease. After intracerebral hemorrhage (ICH), mechanical compression by hematoma, neuroinflammation, oxidative stress, and cytotoxicity of hematoma lysates caused the destruction of the blood brain barrier (BBB). Targeting BBB is a major therapeutic method for patients with ICH. The purpose of the present study was to explore the role of VPA in preserving BBB integrity in the ICH model and investigate the underlying molecular mechanisms. One hundred and thirty-six adult male CD1 mice were randomly divided into five groups in the study. Mice subjected to ICH were administered intraperitoneally with VPA at 3, 24, and 48 h post-ICH, respectively. Neurobehavioral assessments, BBB permeability, Evans blue fluorescence, hematoma volume, and protein expression were evaluated. The administration of VPA reduced BBB permeability and improved the neurobehavior significantly post-ICH. VPA administration significantly decreased the expression of phosphorylated nuclear factor-kappa B (p-NF*κ*B), matrix metalloproteinases 9 (MMP9), tumor necrosis factor*α* (TNF*α*), and interleukin-6 (IL-6), while it enhanced the expression of claudin 5 and occludin in the brain. In conclusion, VPA administration maintained the integrity of BBB after experimental ICH, thus reducing brain edema and improving the neurological outcomes. Therefore, VPA administration might be a new therapeutic method to protect BBB integrity for patients with ICH.

## 1. Introduction

In many ICH patients, the symptoms of neurological deficits continue to worsen gradually, even after the removal of hematoma in many patients [1]. This indicates that the secondary injury of brain tissue is the main factor of aggravation and poor prognosis after ICH. Many mechanisms underlying secondary injury after ICH need to be further explored. Among these, BBB damage is one of the key factors.

In recent years, several studies have confirmed that VPA protects the nervous system through a variety of signaling pathways. It is a traditional antiepileptic drug, and its antiepileptic mechanism is related to the decreased neuronal excitability caused by a variety of comprehensive factors [2]. VPA mainly acts on the gamma aminobutyric acid (GABA) metabolic pathway [3], as well as reduces the levels of glutamate and aspartic acid in the brain [4]. In addition, VPA acts on different ion channels to increase the amplitude and threshold of the action potential [5]. Reportedly, VPA significantly inhibits the toxicity of hemin and reduces nerve cell death in both cortical cells and the brain through decreasing heme oxygenase-1 (HO-1) protein expression [6], neuroinflammation, and perihematomal cell death in the ICH model via transcriptional activation following histone deacetylase (HDAC) inhibition [7]. However, the protective effect and mechanism of VPA on BBB injury after ICH are yet to be elucidated.

Based on the current research and treatment status of the intracerebral hemorrhage, this study is aimed at clarifying the protective effect and potential mechanism of VPA on BBB integrity after ICH and supplying a prospective option for the patients with ICH.

## 2. Materials and Methods

### 2.1. ICH Models

One hundred and thirty-six mice (CD1, male, 35-40 g, 8 weeks old; Vital River, Beijing, China) were needed. All the animals were housed in a room under temperature and humidity control for at least 3 days before ICH surgery and supplied with unlimited food or water. All experiments performed complied with the Chinese Council on Animal Care guidelines and were approved by the Animal Care and Use Committee of Tianjin Medical University (China). The manuscript followed the ARRIVE guidelines.

### 2.2. Experimental Design

All animals were arbitrarily assigned to the following five experiments (Supplementary file 1). The experimental grouping and animal numbers are shown in the supplementary materials (Supplementary file 2).

### 2.3. Experiment 1

Thirty mice were randomly and equally assigned to the sham group, the ICH group, the ICH + VPA (100 mg/kg) group, the ICH + VPA (300 mg/kg) group, and the ICH + VPA (600 mg/kg) group. The neurobehavioral tests and brain water content (BWC) were assessed at 72 h post-ICH.

### 2.4. Experiment 2

Evans blue (EB) extravasation was used to evaluate the effect of VPA on BBB permeability. In this study, eighteen mice were arbitrarily and equally assigned into three groups: the sham group, the ICH group, and the ICH + VPA (300 mg/kg) group. In order to observe the integrity of vascular endothelial cells, immunofluorescence staining was performed at 72 h post-ICH, and nine mice were assigned equally into 3 groups: the sham group, the ICH group, and the ICH + VPA (300 mg/kg) group. To evaluate EB fluorescence at 72 h post-ICH, an additional 9 mice were equally divided into 3 groups: the sham group, the ICH group, and the ICH + VPA (300 mg/kg) group.

### 2.5. Experiment 3

To investigate the effects of VPA on hematoma volume, eighteen mice were randomly assigned into 3 groups: the sham group, the ICH group, and the ICH + VPA (300 mg/kg) group. The hematoma volume was measured at 72 h post-ICH.

### 2.6. Experiment 4

To demonstrate the protection mechanism of VPA on BBB, eighteen mice were equally assigned into the sham group, the ICH group, and the ICH + VPA (300 mg/kg) group. Western blot was performed at 72 h after surgery.

### 2.7. Experiment 5

To evaluate the roles of VPA in long-term neurological functions, twenty-four mice were equally assigned into the sham group, the ICH group, and the ICH + VPA (300 mg/kg) group. On weeks 1, 2, and 3 post-ICH, the foot fault test and the rotarod test were used. On days 21–25 after ICH, the Morris water maze test was performed.

### 2.8. ICH Model

The ICH models in mice were performed by bacterial collagenase injection into the right basal ganglia, as reported previously [8]. The operation procedures in sham mice were performed similarly but only PBS was injected.

### 2.9. Drug Administration

VPA was dissolved into doses of 100, 300, and 600 mg/kg, respectively, with normal saline (0.9%), which were administered at 3, 24, and 48 hours post-ICH intraperitoneally. An equal amount of normal saline was injected in sham mice.

### 2.10. Short-Term Neurobehavioral Tests

Short-term neurobehavioral tests were assessed at 72 hours post-ICH, as reported previously [9, 10].

### 2.11. BWC Assessment

BWC was performed at 72 hours post-ICH using the wet/dry method, as reported previously [11]. The calculation formula of brain water content (%) is as follows: (wetweight − dryweight)/wetweight × 100%.

### 2.12. Blood Brain Barrier Permeability

EB dye extravasation and immunofluorescence staining were used to evaluate BBB permeability using spectrophotometry and fluorescence microscopy at 72 hours after ICH induction, as described previously [12, 13]. 4% EB dye solution was injected intraperitoneally and circulated in vivo for at least 3 h [14]. EB dye absorbance was measured by spectrophotometry (Stech, Shanghai, China) and quantified with a standard curve at 610 nm.

Immunofluorescence staining was conducted at 72 h following ICH as described previously [15]. Primary antibodies used were claudin 5 (1 : 200, Santa Cruz Biotechnology) and Von Willebrand Factor (VWF) (1 : 400, ab6994). For EB fluorescence, mice were intraperitoneally injected with 4% EB dye solution. The dye was allowed to circulate for 3 h, and then the mice underwent intracardiac perfusion under deep anesthesia with PBS and paraformaldehyde. Brain tissues were separated and frozen for 24 hours at -80°C. The coronal slices (8 *μ*m) were collected with a cryostat (KD-2950; Jinhua Kedi Instrumental Equipment Co., Ltd., Jinhua, China). The red autofluorescence of the EB dye was observed by rhodamine fluorescence excitation and emission filters (Leica Microsystems). The fluorescence intensity of five random sections of the ipsilateral cortex was analyzed in each slide.

### 2.13. Hematoma Volume Assessment

Hematoma volume was detected at 24 hours post-ICH by hemoglobin assay, as described previously [14]. The absorbance of hemoglobin was determined at 540 nm with a spectrophotometer.

### 2.14. Western Blot

Western blot analysis was performed as described previously [16, 17]. Brain sections were collected at 72 hours post-ICH. Primary antibodies used are as follows: NF*κ*B (1 : 2000, ab16502), p-NF*κ*B (1 : 500, ab86299), MMP9 (1 : 1000, ab38898), TNF*α* (1 : 1000, ab6671), IL-6 (1 : 1000, ab6672), claudin 5 (1 : 400, Santa Cruz Biotechnology), and occludin (1 : 50000, ab216327). *β*-Actin (1 : 1000, Santa Cruz Biotechnology) served as an internal loading control. The bands were quantified by densitometry with ImageJ software (ImageJ 1.4, NIH, USA).

### 2.15. Long-Term Neurobehavioral Tests

In the long-term neurobehavioral tests, in order to assess sensorimotor function, coordination, and balance of animals, the foot fault test and the rotarod test were used at weeks 1, 2, and 3 post-ICH, as described previously [18]. Morris water maze tests, consisting of escape latency and swim distance, were conducted on 21–25 days post-ICH to assess memory function and spatial learning capacity as described previously [19].

### 2.16. Statistical Analysis

The data analysis was conducted with GraphPad Prism 6 software. Data are expressed as mean ± SD. Statistical differences were analyzed between groups with one-way ANOVA, followed by Tukey's post hoc tests. *p* values < 0.05 was considered to be statistically significant.

## 3. Results

### 3.1. Animals

No mice died in the sham group. Of the 98 surgeries, 8 mice died in this study due to serious hematoma. The total mortality of the study was 8.16% (8/98). Two mice with mild hemorrhage were excluded from this study (Supplementary file 2).

### 3.2. VPA Treatment Improved Neurological Functions and Reduced BWC at 72 Hours Post-ICH

**Figure 1:**
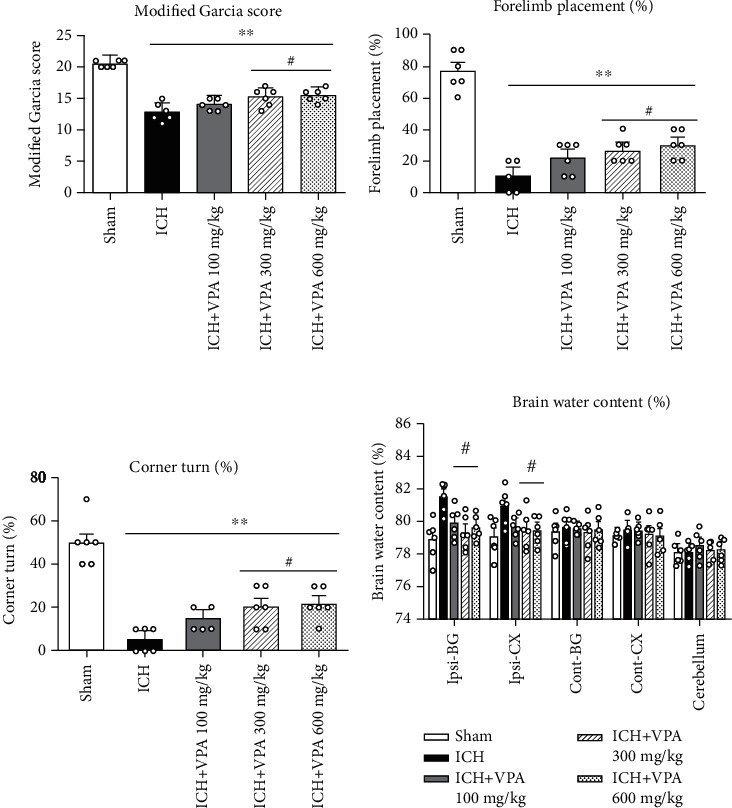
Medium and high doses of VPA treatment improved the neurobehavioral functions, including the modified Garcia score (a), the forelimb placing test (b), and the corner turn test (c), and they reduced brain water content (BWC) (d) at 72 h after ICH. ^#^*p* < 0.05 versus ICH; ^∗^*p* < 0.05 and ^∗∗^*p* < 0.01 versus sham. Ipsi-BG: ipsilateral basal ganglia; Ipsi-CX: ipsilateral cortex; Cont-BG: contralateral basal ganglia; Cont-CX: contralateral cortex.

At 72 h post-ICH, mice displayed significantly worse outcome compared with the sham group in the neurobehavioral tests. The medium and high doses of VPA treatment remarkably improved the neurobehavioral outcome and reduced BWC in the ipsilateral hemisphere compared with that in the ICH group (Figures 1(a)–1(d)). Then, the medium dose of VPA was selected in the next experiments.

### 3.3. VPA Treatment Attenuated BBB Permeability after ICH

**Figure 2:**
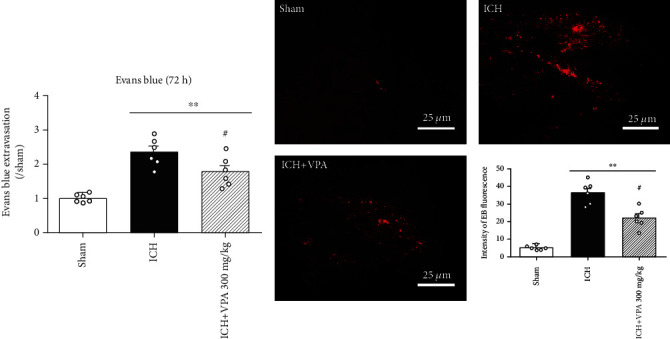
(a) VPA administration decreased EB dye accumulation at 72 h after ICH. ICH significantly increased EB dye accumulation in the ipsilateral hemisphere. However, VPA treatment remarkably reversed the result. (b) Typical fluorescent micrographs of EB extravasation and quantitative analysis of EB fluorescence intensity in the ipsilateral cortex. ^#^*p* < 0.05 versus ICH; ^∗∗^*p* < 0.01 versus sham. Scalebar = 25*μ*m.

**Figure 3:**
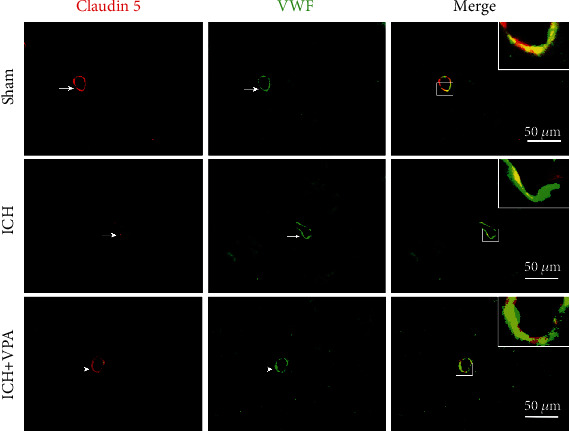
ICH significantly decreased the expression of claudin 5 in vascular endothelial cells in the ipsilateral hemisphere. However, administration of VPA increased the expression of claudin 5 in vascular endothelial cells at 72 hours post-ICH. Scalebar = 50*μ*m.

There was EB dye accumulated in the ipsilateral hemisphere of mice in the ICH group at 72 hours after ICH surgery. VPA treatment significantly attenuated the ICH-induced dye accumulation in comparison to the ICH group (Figure 2(a)). The intensity of EB fluorescence was consistent with the EB extravasation findings in the ipsilateral cortex (Figure 2(b)). Immunofluorescence staining showed that the claudin 5 expression in vascular endothelial cells was decreased significantly in the ICH group as compared to the sham group. However, claudin 5 expression was increased significantly after VPA treatment (Figure 3).

### 3.4. VPA Treatment Did Not Decrease Hematoma Volume at 72 h Post-ICH

**Figure 4:**
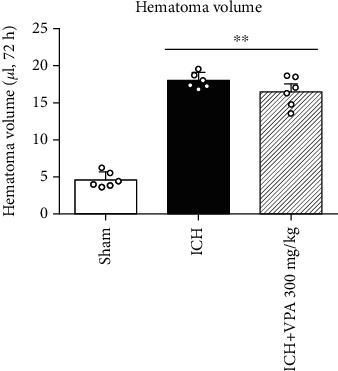
Injection of collagenase resulted in hematoma in the ICH and ICH + VPA groups. VPA treatment (300 mg/kg) did not reduce the hematoma volume compared to the ICH group (*p* > 0.05). ^∗∗^*p* < 0.01 versus sham.

Injection of collagenase induced ICH in the ipsilateral hemispheres. The hematoma volume was smaller in the VPA treatment group as compared to the ICH group at 72 h post-ICH, but there was no statistical difference (Figure 4).

### 3.5. Effect of VPA on the Downstream Signaling Pathway Post-ICH

**Figure 5:**
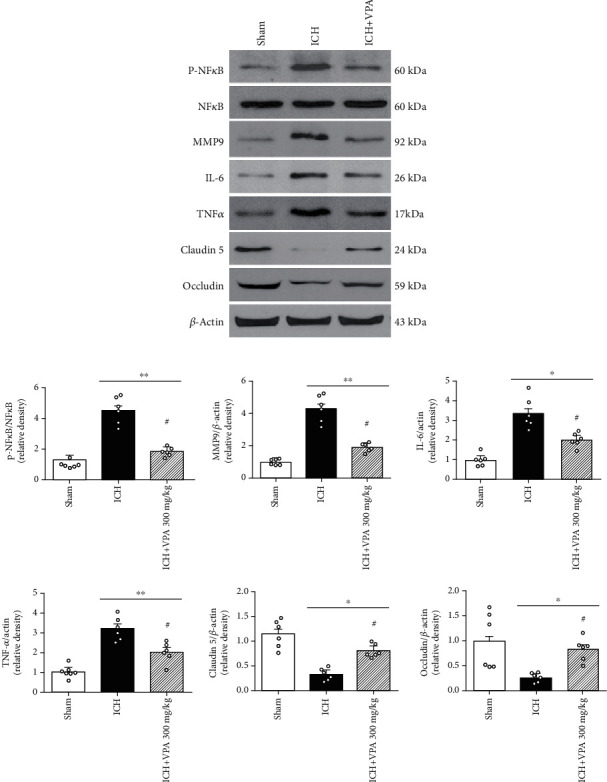
Effects of VPA on the expression of downstream signal pathway proteins post-ICH. (a) Representative bands of the protein expressions in western blot. (b) Quantitative analysis of p-NF*κ*B, MMP9, IL-6, TNF*α*, occludin, and claudin 5 at 72 h post-ICH. ^#^*p* < 0.05 versus ICH; ^∗^*p* < 0.05 and ^∗∗^*p* < 0.01 versus sham.

At 72 hours after ICH, the expressions of p-NF*κ*B, MMP9, IL-6, and TNF*α* were increased, whereas the endothelial junction proteins were remarkably reduced in the ICH group. However, VPA administration reversed the above results (Figures 5(a) and 5(b)).

### 3.6. VPA Ameliorated Long-Term Neurological Impairments Post-ICH

**Figure 6:**
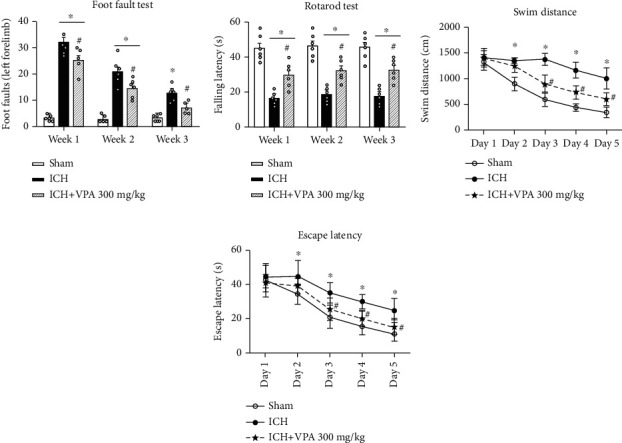
The long-term neurobehavioral impairments were improved in ICH mice after VPA administration. (a, b) VPA treatment reduced the frequency of foot faults and prolonged the latency to fall on weeks 1, 2, and 3 after ICH. (c, d) VPA treatment improved the performance of mice on days 23–25 after ICH in the Morris water maze test. ^#^*p* < 0.05 versus ICH; ^∗^*p* < 0.05 versus sham.

Animals from the ICH group demonstrated significantly higher foot faults in the foot fault test and a shorter latency to fall in the rotarod test. However, VPA treatment changed the results (Figures 6(a) and 6(b)). Furthermore, the ICH group mice performed worse in swim distance and escape latency in the water maze test. However, VPA administration significantly ameliorated the outcomes (Figures 6(c) and 6(d)).

## 4. Discussion

In the current study, we demonstrated that VPA treatment improved BBB integrity in the ICH model, which was accompanied by a decrease in the activity of p-NF*κ*B, MMP9, IL-6, and TNF*α* as well as an increase in the level of endothelial junction proteins, which in turn, ameliorated short-term and long-term neurological impairments after ICH. Together, these findings suggested that VPA administration could preserve BBB integrity, thereby improving the outcomes after ICH. The protective effects of VPA on BBB might be effectuated via the inhibition of p-NF*κ*B and MMP9 and upregulation of endothelial junction protein factors claudin 5 and occludin. The schematic of the mechanism is displayed in supplementary file 3.

ICH is a serious stroke subtype with extensive BBB disruption characteristics [20]. Many studies have confirmed that damaged BBB is crucially related to adverse prognosis after ICH. The pathological mechanism underlying BBB destruction after ICH mainly includes mechanical compression of brain tissue by hematoma, inflammation, ischemia of local brain tissue, oxidative stress, thrombin toxicity, and cytotoxicity of hematoma lysates [21–24]. The structure and energy of BBB depend on the interaction of cellular and acellular components [25, 26]. The treatment of increasing the expression of tight junction proteins can ameliorate brain edema and injury in ICH mice [27]. In our study, VPA administration resulted in a dramatic increase in the level of tight junction proteins, a reduction in BWC, and a decrease in BBB permeability as EB dye extravasation and fluorescence were reduced in comparison to the ICH group, which coincided with the improvement in neurological functions.

VPA is one of the most widely used antiepileptic drugs, which can alleviate the damage of neurons associated with epileptic activities. Notably, the antiepileptic effect of VPA is due to its multitarget influence in the central nervous system, including the reduction of N-methyl-D-aspartic acid-mediated neuronal excitation, inhibition of GABA transamination, and inhibition of glycogen synthase kinase-3 activity [28]. VPA is an inhibitor of histone deacetylase I (1, 2, 3, and 8 isoforms) and IIa (4, 5, 7, and 9 isoforms) [29]. As an HDAC inhibitor, VPA causes histone hyperacetylation, chromatin relaxation, and gene transcription, and it exerts a variety of neuroprotective roles. It has been confirmed that VPA upregulates alpha-synuclein by inhibiting HDAC, thus inhibiting glutamate neurotoxicity in rat cerebellar granular cells [30]. In the experimental model of transient or permanent focal cerebral ischemia in rats, VPA treatment restores the reduction in histone acetylation; inhibits the downregulation of phosphorylated AKT, activation of caspase-3, and overexpression of p53; alleviates neuronal apoptosis; reduces infarct volume; and improves neurological deficit [31, 32].

Although VPA has been used in many models, the optimal dosage of VPA is yet to be deduced. 200 mg/kg VPA is known to exert neuroprotective effects by reducing hippocampal neuron loss and improving cognitive function after cerebral ischemia in rats [33]. A previous study showed that the treatment of VPA (300 mg/kg) significantly reduced the growth of brain lesions with respect to delayed cerebral ischemia after subarachnoid hemorrhage [34]. Hemorrhage volume is a key factor related to most accepted predictors of ICH prognosis [35]. Sinn et al. demonstrated that after a higher dose of VPA (300 mg/kg, twice a day) treatment, the hematoma volume was significantly reduced as compared to the ICH group [7]. An interesting fact is that VPA is involved in the regulation of brain protection in a dose-dependent manner after traumatic brain injury [36]. VPA-mediated BBB protection was positively correlated with the dose of VPA and lasted for at least 3 days after cerebral ischemia and reperfusion [37]. The current results showed that medium-dose and high-dose VPA increased the short-term neurobehavioral function and BWC at 72 hours in ICH mice. Furthermore, there was no significant difference in medium- and high-dose groups. Therefore, we chose the medium dose of VPA in the subsequent experiments. However, VPA did not significantly reduce the hematoma size at 72 h after ICH. The findings implied that the protective effect of VPA on BBB might be closely related to the dose. The optimal administration time of VPA has not yet been determined. Reportedly, the injection of VPA before or immediately after ischemia can reduce the infarction area in mice with transient middle cerebral artery occlusion (tMCAO). However, VPA treatment at 4 h after reperfusion does not improve the neurological deficit [38]. Another study showed that VPA treatment 24 hours after MCAO for 7 days promotes neurogenesis and leukoencephalopath recovery and improves the prognosis of pMCAO rats [39]. The inconsistencies between the two studies may be due to different models and dosages.

Matrix metalloproteinases (MMPs) are a family of zinc ion- and calcium ion-dependent proteases, which can degrade the extracellular matrix. The expression level of MMPs in the normal adult brain is low beyond the detection level but can be increased when tumor invasion and metastasis, inflammatory reaction, vascular regeneration, and other pathological processes occur. Rosenberg found that MMP9 expression reaches to the highest peak at 24 hours after ICH induced by collagenase [28]. Several studies have confirmed that MMP9 damages the integrity of the cell basement membrane, increases the permeability of BBB, and aggravates brain edema by inducing inflammatory cascade reaction and calcium ion overload [40, 41]. Animal experiments also showed that the expression of MMP9 was detected in both lesion and surrounding areas, indicating that the molecule is involved in the growth of the ischemic area [40]. Therefore, targeting MMP9 might provide a practical clinical strategy for the patients with brain injury.

Reportedly, VPA can reduce the expression of MMP9 and the degradation of endothelial tight junction proteins and nuclear translocation of NF*κ*B; protect the integrity of BBB function and structure; and alleviate brain edema and inflammation by restoring histone acetylation in the rat MCAO model [41, 42]. Therefore, the protection of BBB by VPA in ischemic animal models might involve the inhibition of HDAC, followed by the inhibition of p-NF*κ*B activation and the overexpression of MMP9, thus exerting the protective effect on BBB. These phenomena were consistent with the results of our study.

Capillary endothelial cells and the tight junctions between adjacent cells are critical parts of BBB. The permeability of BBB is closely associated with the function and structure of tight junctions [43]. Under various pathological conditions, the tight junction protein performs a vital role in maintaining BBB integrity [44, 45]. Moreover, tight junction proteins can form a selectively permeable barrier and seal the intercellular space of adjacent endothelial cells to circulating molecules. According to the current data, ICH decreases the expression of occludin and claudin 5, while VPA treatment reverses the result.

In recent years, the neuroprotective effect of VPA has been confirmed in several models, including stroke, epilepsy, and traumatic brain injury. VPA reduces brain injury via anti-inflammation, anti-neuronal apoptosis, and neurotrophic effects. In the tMCAO rat model, VPA decreased the activation of caspase-3, reduced the area of cerebral infarction, and enhanced functional recovery. The beneficial effect of VPA might be based on the inhibition of HDAC and upregulation of heat shock protein 70 induced by VPA [32]. In the ICH mice model, VPA inhibits the neuroinflammation of perihematoma, reduces the quantity of neuron apoptosis, prevents hematoma expansion, and improves the neurological deficit [7]. Another study showed that VPA administration enhances ubiquitination and proteasome degradation of HO-1 protein by activating the ERK1/2 and JNK signaling pathways, thus partially alleviating hemin toxicity after ICH [6]. However, the potential mechanism of VPA-mediated protection of BBB integrity after ICH remains unclear. In this study, we demonstrated that after 72 h of VPA treatment, BBB permeability was dramatically decreased, and also, the neurological deficit was improved. Western blot assay revealed that the overexpression of p-NF*κ*B, MMP9, IL-6, and TNF*α* induced by ICH in VPA-treated mice was markedly reduced and the expression of occludin and claudin 5 was significantly increased. As far as we know, this is the first study where VPA has been used to protect BBB in the ICH model.

Nevertheless, the present study has some limitations. VPA may exert a variety of protective effects through various signaling pathways in the central nervous system [34, 36]. In the present study, we only pay attention to the effect of VPA on BBB. Therefore, we are unable to eliminate the possibilities of antiapoptotic, anti-inflammatory, antioxidant stress, and synaptic plasticity effects of VPA in brain injury after ICH. Second, although VPA (300 mg/kg) had no effect on the hematoma size induced by collagenase at 72 h after ICH, different results may be obtained with prolonged observation or an increased VPA dose. Therefore, it is necessary to investigate the reasonable dose of VPA in the ICH model in the future.

In conclusion, the administration of VPA after ICH improves neurological impairment, reduces BWC, and preserves the integrity of BBB in mice. This neuroprotection effect of VPA is achieved by inhibiting the activation of the NF*κ*B/MMP9 signaling pathway. Therefore, VPA might be a potential effective therapy for BBB protection in ICH patients.

## Data Availability

The data, analytic methods, and study materials are available for reproducing the results or replicating the procedures. The data that support the findings of this study are available from the corresponding author upon reasonable request. The authors will be responsible for maintaining availability.

## Supplementary Materials

Supplementary 1 Supplementary file 1: the experimental design. To evaluate the effects of VPA on BBB in ICH mice, this experimental design included five parts. Mice subjected to ICH were administered intraperitoneally with VPA at 3, 24, and 48 h after ICH, respectively. Neurobehavioral tests, BWC, EB, hematoma volume, and WB were evaluated after ICH.

Supplementary 2 Supplementary file 2: summary of experimental groups and mortality rate in the study. All animals were arbitrarily assigned into five experiments. The summary of the experimental groupings, the number of animals, and the mortality rates in experiments 1–5 are listed and shown. A total of 136 mice were used of which 38 were sham and 98 mice underwent ICH induction. None of the sham mice died, and the total mortality rate in the ICH group was 8.16% (8/98).

Supplementary 3 Supplementary file 3: the schematic of the mechanism. In the nucleus, histone and DNA form a DNA-histone complex, and the change of the conformation of the complex regulates the transcription of the DNA. In normal conditions, two kinds of proteases, histone acetylases (HATs) and histone deacetylases (HDACs), keep the DNA-histone complex in equilibrium. The histone acetylation catalyzed by HATs can promote the dissociation of DNA and histone, so transcription factors can bind to DNA binding sites specifically, thus facilitating gene transcription. In general, the addition of acetyl groups to histones by HATs is correlated with an increase in gene expression, whereas the removal of acetyl groups by HDACs is associated with transcriptional repression. Therefore, as a HDAC inhibitor, VPA can increase the level of histone acetylation, inhibit the activation of NF*κ*B caused by ICH, decrease the expression of MMP9, upregulate the expression of tight junction proteins, and protect the integrity of BBB.
