# Supplementary material for: Valproate Sodium Protects Blood Brain Barrier Integrity in Intracerebral Hemorrhage Mice
Source: Oxid Med Cell Longev. 2020 Nov 10;2020:8884320. doi: 10.1155/2020/8884320 (PMC7676278; doi:10.1155/2020/8884320)
Supplement: Supplementary 1 — Supplementary file 1: the experimental design. To evaluate the effects of VPA on BBB in ICH mice, this experimental design included five parts. Mice subjected to ICH were administered intraperitoneally with VPA at 3, 24, and 48 h after ICH, respectively. Neurobehavioral tests, BWC, EB, hematoma volume, and WB were evaluated after ICH. [file 8884320.f1.docx]

**Supplementary file 1: The experimental design**


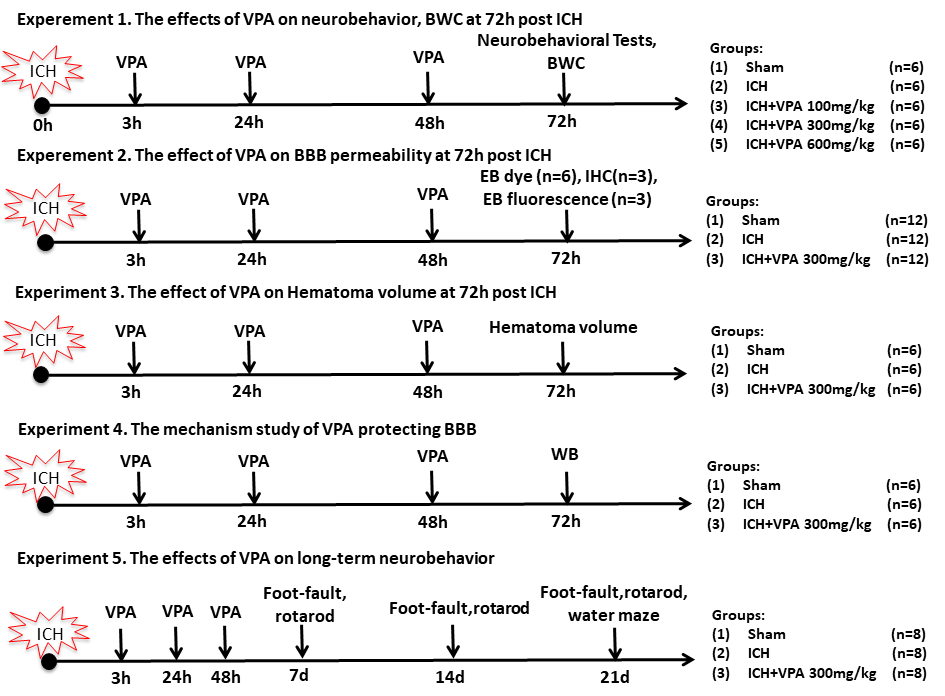


Abbreviation: ICH, intracerebral hemorrhage; VPA, valproate sodium; BWC, brain water content; EB, evans blue; IHC, immunohistochemistry; WB, western blot

**Experiment 1:** To evaluate the effects of VPA on neurobehavioral tests and brain water content (BWC) at 72 h after post ICH, 30 mice were randomly and equally assigned divided into 5 groups (n=6): Sham, ICH, ICH + VPA (100 mg/kg), ICH + VPA (300 mg/kg), and ICH + VPA (600 mg/kg). VPA was injected intraperitoneally administered at 3, 24, and 48 h post-ICH respectively.

**Experiment 2:** To assess the effects of VPA for BBB permeability, Evans blue (EB) extravasation was evaluated at 72 h post-ICH. 18 mice were randomly and equally assigned into 3 groups (n=6): Sham, ICH, and ICH + VPA (300 mg/kg). To observe the integrity of vascular endothelial cells, immunofluorescence staining was performed at 72 h post-ICH, 9 mice assigned equally into 3 groups: Sham, ICH, and ICH + VPA (300 mg/kg). To evaluate EB fluorescence at 72 h post ICH, additional 9 mice were equally assigned into 3 groups: Sham, ICH, and ICH + VPA (300 mg/kg). VPA was administered intraperitoneally at 3, 24, and 48 h post-ICH respectively.

**Experiment 3:** To evaluate the effect of VPA on hematoma volume at 72 h post-ICH, 18 mice were randomly and equally assigned into 3 groups (n=6): Sham, ICH, and ICH + VPA (300 mg/kg). VPA was intraperitoneally administered at 3, 24, and 48 h post-ICH respectively.

**Experiment 4:** To verify the protection mechanism of VPA on BBB, western blot (WB) was performed at 72 h after post-ICH. 18 mice were assigned divided equally into 3 groups (n=9): Sham, ICH, and ICH + VPA (300 mg/kg). VPA was intraperitoneally administered at 3, 24, and 48 h post-ICH respectively.

**Experiment 5:** To evaluate the roles of VPA in long-term neurological functions, 24 mice were equally assigned into 3 groups: Sham, ICH, and ICH + VPA (300 mg/kg). VPA was intraperitoneally administered at 3, 24, and 48 h post-ICH respectively. On weeks 1, 2, and 3 post-ICH, foot fault test and rotarod test were used. On days 21–25 after ICH, Morris water maze test was performed.
