# Supplementary material for: Valproate Sodium Protects Blood Brain Barrier Integrity in Intracerebral Hemorrhage Mice
Source: Oxid Med Cell Longev. 2020 Nov 10;2020:8884320. doi: 10.1155/2020/8884320 (PMC7676278; doi:10.1155/2020/8884320)
Supplement: Supplementary 2 — Supplementary file 2: summary of experimental groups and mortality rate in the study. All animals were arbitrarily assigned into five experiments. The summary of the experimental groupings, the number of animals, and the mortality rates in experiments 1–5 are listed and shown. A total of 136 mice were used of which 38 were sham and 98 mice underwent ICH induction. None of the sham mice died, and the total mortality rate in the ICH group was 8.16% (8/98). [file 8884320.f2.docx]

| **Experimental**  **Groups** | **Neurobehavior test**  **BWC** | **EB**  **fluorescence** | **EB dye** | **IHC** | **Hematoma**  **volume** | **WB** | **Exclusion** | **Mortality**  **(%)** | **Subtotal** |
| --- | --- | --- | --- | --- | --- | --- | --- | --- | --- |
| **Experimental 1** |  |  |  |  |  |  |  |  |  |
| Sham | 6 |  |  |  |  |  | 0 | 0 | 6 |
| ICH | 6 |  |  |  |  |  | 1 | 1(14.29%) | 7 |
| ICH+VPA100mg/kg | 6 |  |  |  |  |  | 0 | 0 | 6 |
| ICH+VPA300mg/kg | 6 |  |  |  |  |  | 0 | 0 | 6 |
| ICH+VPA600mg/kg | 6 |  |  |  |  |  | 1 | 1(14.29%) | 7 |
| **Experimental 2** |  |  |  |  |  |  |  |  |  |
| Sham |  | 3 | 6 | 3 |  |  | 0 | 0 | 12 |
| ICH |  | 3 | 6 | 3 |  |  | 2 | 1(7.14%) | 14 |
| ICH+VPA300mg/kg |  | 3 | 6 | 3 |  |  | 0 | 0 | 12 |
| **Experimental 3** |  |  |  |  |  |  |  |  |  |
| Sham |  |  |  |  | 6 |  | 0 | 0 | 6 |
| ICH |  |  |  |  | 6 |  | 1 | 1(14.29%) | 7 |
| ICH+VPA300mg/kg |  |  |  |  | 6 |  | 1 | 1(14.29%) | 7 |
| **Experimental 4** |  |  |  |  |  |  |  |  |  |
| Sham |  |  |  |  |  | 6 | 0 | 0 | 6 |
| ICH |  |  |  |  |  | 6 | 2 | 1(12.5%) | 8 |
| ICH+VPA300mg/kg |  |  |  |  |  | 6 | 0 | 0 | 6 |
| **Experimental 5** |  |  |  |  |  |  |  |  |  |
| Sham | 8 |  |  |  |  |  | 0 | 0 | 8 |
| ICH | 8 |  |  |  |  |  | 1 | 1(11.1%) | 9 |
| ICH+VPA300mg/kg | 8 |  |  |  |  |  | 1 | 1(11.1%) | 9 |
| **Total** | 54 | 9 | 18 | 9 | 18 | 18 | 10 | 8(8.16%) | 136 |

Supplementary file 2: summary of experimental groups and mortality rate in the study

ICH, intracerebral hemorrhage; VPA, valproate sodium; BWC, brain water content; EB, evans blue; IHC, immunohistochemistry; WB, western blot

All animals were arbitrary assigned into the five experiments. The summary of experimental groupings, the number of animals, and mortality rate in experiments 1–5 are listed showed in this table. A total of 136 mice were used of which 38 were sham and 98 mice underwent ICH induction. None of the sham mice died and the total mortality rate in ICH group was 8.16% (8/98).
