# Supplementary material for: Valproate Sodium Protects Blood Brain Barrier Integrity in Intracerebral Hemorrhage Mice
Source: Oxid Med Cell Longev. 2020 Nov 10;2020:8884320. doi: 10.1155/2020/8884320 (PMC7676278; doi:10.1155/2020/8884320)
Supplement: Supplementary 3 — Supplementary file 3: the schematic of the mechanism. In the nucleus, histone and DNA form a DNA-histone complex, and the change of the conformation of the complex regulates the transcription of the DNA. In normal conditions, two kinds of proteases, histone acetylases (HATs) and histone deacetylases (HDACs), keep the DNA-histone complex in equilibrium. The histone acetylation catalyzed by HATs can promote the dissociation of DNA and histone, so transcription factors can bind to DNA binding sites specifically, thus facilitating gene transcription. In general, the addition of acetyl groups to histones by HATs is correlated with an increase in gene expression, whereas the removal of acetyl groups by HDACs is associated with transcriptional repression. Therefore, as a HDAC inhibitor, VPA can increase the level of histone acetylation, inhibit the activation of NFκB caused by ICH, decrease the expression of MMP9, upregulate the expression of tight junction proteins, and protect the integrity of BBB. [file 8884320.f3.docx]

**Supplemental file 3:** **The schematic of the mechanism in the study**


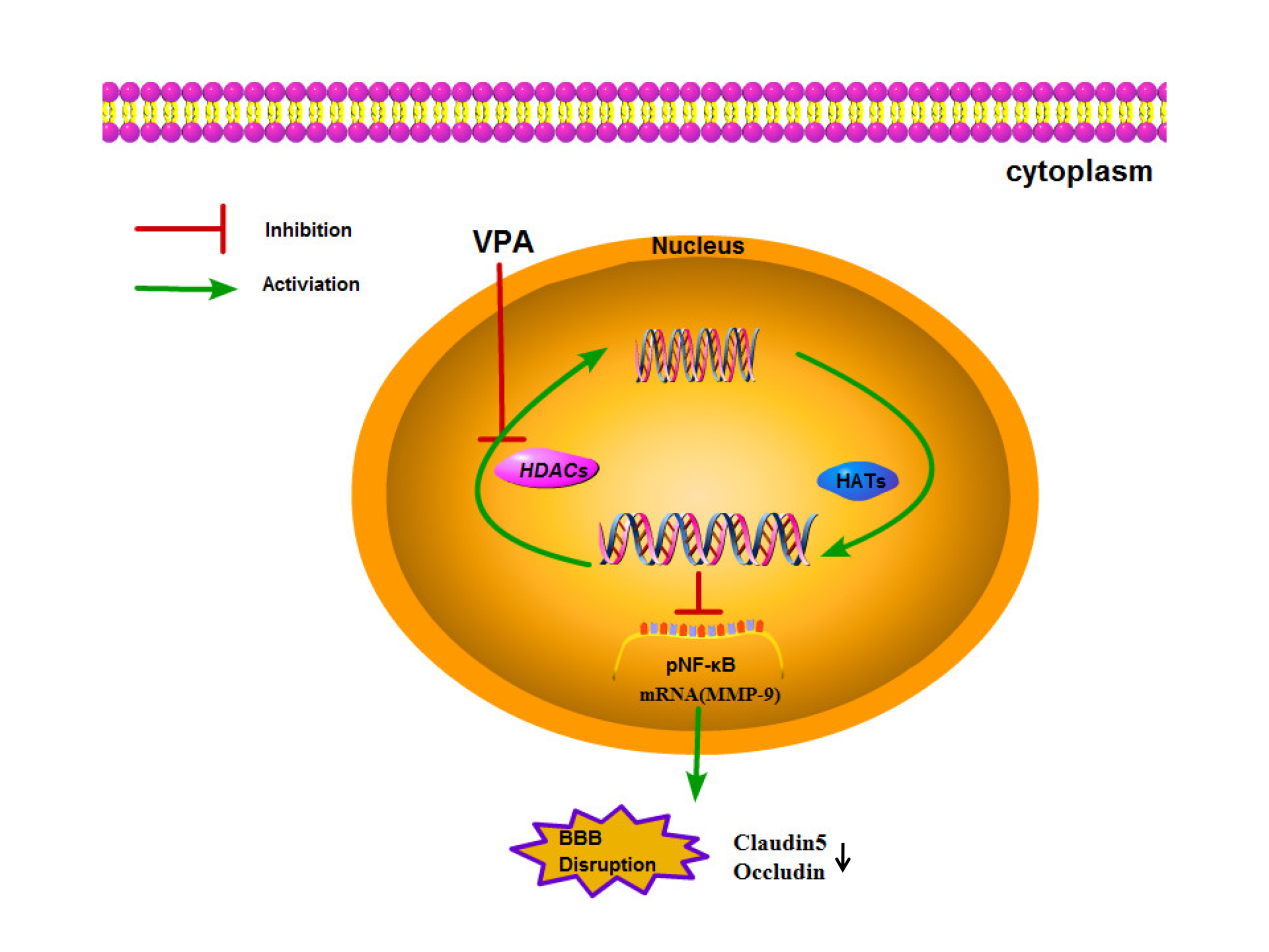


In the nucleus, histone and DNA form a DNA-histone complex, the change of the conformation of the complex regulates the transcription of the DNA. In normal condition, two kinds of protease, histone acetylases (HATs) and histone deacetylases (HDACs), keep the DNA-histone complex in equilibrium. The histone acetylation catalyzed by HATs can promote the dissociation of DNA and histone, so transcription factors can bind to DNA binding sites specifically, thus facilitating gene transcription. In general, the addition of acetyl groups to histones by HATs is correlated with an increase in gene expression, whereas removal of acetyl groups by HDACs is associated with transcriptional repression. Therefore, as a HDACs inhibitor, VPA can increase the level of histone acetylation, inhibit the activation of NFκB caused by ICH, decrease the expression of MMP9, up regulate the expression of tight junction protein, and protect the integrity of BBB.
